# The effect of biogas slurry application on biomass production and the silage quality of corn

**DOI:** 10.5713/ab.23.0129

**Published:** 2023-08-23

**Authors:** Hua Sun, Kai Shi, Hairong Ding, Chenglong Ding, Zhiqing Yang, Chen An, Chongfu Jin, Beiyi Liu, Zhaoxin Zhong, Xia Xiao, Fuyin Hou

**Affiliations:** 1 Jiangsu Coastal Area Institute of Agricultural Science, Yancheng 224000, China; 2 Institute of Animal Science, Jiangsu Academy of Agricultural Sciences, Nanjing 210014, China

**Keywords:** Biogas Slurry, Biomass Yield, Corn Silage, Fermentation Quality, Forage Quality

## Abstract

**Objective:**

The objective of this study was to evaluate the effect of biogas slurry application on biomass production and the silage quality of corn.

**Methods:**

A field experiment was conducted in which corn was grown using different biogas slurry application rates. The effect of 25% to 500% biogas slurry nitrogen replacement (T1 to T14) on the yield and quality indices of corn were studied by field plot experiments.

**Results:**

The results revealed that biogas slurry application improved the stem diameter and relative feed value of corn silage in treatments T13 and T11. Moreover, the fermentation quality of corn silage was improved due to an increase in lactic acid content; in comparison with the chemical synthetic fertilizer (CF) group. The crude protein contents of corn silage had no obvious change with increasing biogas slurry application. However, the forage quality index of acid detergent fiber was decreased (p<0.05) in the T11 group compared with the CF group. In addition, higher (p<0.05) 30 h *in vitro* dry matter digestibility and 30 h *in vitro* neutral detergent fiber digestibility were observed in the T11 and T13 groups than in the CF group.

**Conclusion:**

Based on these results, it was concluded that the optimum biogas slurry application rate for corn was approximately 350% to 450% biogas slurry nitrogen replacement under the present experimental conditions.

## INTRODUCTION

As the numbers of livestock have increased all around the world, the amount of animal manure has simultaneously increased. In China, the population of dairy cows is more than 10 million [1]. A few more than 100 dairy cattle factories account for approximately 60% of the dairy population, and the proportion of large-scale dairy cattle farming has been increasing. With increases in the number of dairy cattle and the scale of operations, massive amounts of livestock wastes, such as feces, urine and washing water from breeding, are produced, which are difficult to dispose, resulting in extensive environmental pollution. Holm-Nielsen et al [2] have suggested that increasing animal production areas must have suitable manure management practices to optimize waste recycling. For sustainable development, it is necessary to determine a cost-effective way to dispose of these wastes.

Anaerobic digestion is an effective solution for livestock waste and satisfies growing concerns regarding energy supply. It was reported that more than 450 million tons of biogas slurry have been used in China each year [3] to achieve circular agriculture, recycle agricultural wastes, reduce chemical fertilizer input, and protect the environment. However, the increasing popularity of anaerobic digestion has created another challenge associated with the disposal of the large quantities of biogas slurry generated during this process. Improper management results in contamination to surrounding surface water, soil, atmosphere, and groundwater. Additionally, this contamination can affect the growth of animals and plants, and finally pose hidden risks to human health through the food chain. As a product of anaerobic fermentation, biogas slurry contains a variety of water-soluble nutrients required by crops. It serves as a kind of fast-acting water and fertilizer with a strong capacity for providing available nutrients and showing a high utilization rate of nutrients that are quickly absorbed by crops [4]. Biogas slurry has abundant amounts nitrogen (N), phosphorus (P), potassium (K), and other trace elements, which can provide nutrients for crops [5]. Furthermore, biogas slurry could be used as a biological pesticide due to its high levels of amino acids, growth hormones, and antibiotics that promote plant growth [6]. Biogas slurry can stimulate plant roots to secrete phosphatase, which can mineralize organic phosphorus and then be absorbed by plants, which can improve soil organic matter content and increase water retention as well as soil fertility to improve soil properties [7]. In addition, the application of biogas slurry can avoid damage to the soil structure that results from the continuous large-scale application of chemical fertilizer, promote the favorable elemental cycling in soil ecosystems, and reduce crop diseases and insect pests [8]. It can also reduce the environmental pollution caused by chemical fertilizer application [9]. It has been reported that biogas slurry can be efficiently utilized for crops and forage. Studies report elevated yields of wheat and rice [10,11], increased grain yield and biomass in corn and peanut [12,13] and improved production of Italian ryegrass [14]. However, different standards are required for various crops based on plant nutrient utilization efficiency, soil conditions and nutrient environmental release properties.

Corn is an important roughage source for ruminant production, with high biological yield, high starch content, high available energy, good silage properties and strong adaptability [15]. Additionally, corn requires more water and fertilizer than other crops, and it is sensitive to nitrogen availability. It is well-known that nitrogen fertilization directly contributes to the quantity and quality of forage production. However, inappropriate, or excessive use of chemical fertilizers induces adverse effects on the soil, causing a decrease in organic carbon, as well as environmental pollution [13]. Recently, researchers have conducted a large number of biogas slurry studies focused on cereal crops or conventional vegetables. However, there are limited data available on the growth, biomass yield, nutrient content, chemical composition, and silage quality of corn treated with different doses of biogas slurry. Our hypothesis was that biogas slurry could be used to improve the yield and quality of corn to relieve pressures associated with domestic dairy manure disposal and a lack of high-quality forage. Therefore, the objective of this study was to evaluate different application rates of biogas slurry on the biomass and quality of corn and find the optimal dose of dissolved biogas slurry.

## MATERIALS AND METHODS

### Experimental materials and procedures

The experiment was carried out in an experimental field at the Jiangsu dairy industry technology system (Yancheng, China), located at N 33°11′ and E 120°21′. The soil parameters of the experimental field were as follows: organic matter 9.09 g/kg, total nitrogen 0.84 g/kg, rapidly available phosphorus 31.25 mg/kg, rapidly available potassium 118 mg/kg, and pH 8.08. The corn cultivar Ruihuayu No. 3 was used as the plant material in the study. Seeds were sown over 3×5 m plots with 30 cm row spacing and 20 cm spacing in the row. Experiments were conducted in a split-split plot experimental design with three replicates. Sixteen different levels of nitrogen replacement treatment with biogas slurry (CK, no nitrogen fertilizer; CF, chemical synthetic fertilizer; T1 = 25%; T2 = 50%; T3 = 75%; T4 = 100%; T5 = 125%; T6 = 150%; T7 = 175%; T8 = 200%; T9 = 250%; T10 = 300%; T11 = 350%; T12 = 400%; T13 = 450%; T14 = 500%) were applied to the plots. Hoeing and chemical weed control were practiced throughout the growing season. Morphologic observations were collected at the flowering, filling, and milk-dough stages of the plants, and then the plants were harvested.

Corn grown under different biogas slurry levels was harvested at the milk-dough stage. The plants were chopped into 2.5 to 3 cm pieces and immediately packed into deflated vacuum bags (28 cm×38 cm; CNON Packing Co. Ltd., Hebei, China) in triplicate. Then, the samples were preserved at ambient temperature for 60 d.

### Biochemical analysis

The sample bags were opened, and a 30 g sample was mixed with 270 mL water to measure the pH of the samples. The dry matter (DM) content of corn before and after ensiling was determined after drying at 70°C for 48 h in an oven with forced air circulation [16]. Dried samples were then ground in a mill, passed through a 1 mm sieve, and prepared for chemical analyses. Crude protein (CP) was analyzed by the Kjeldahl method (AOAC, 2005; method 990.03) using a Kjeldahl nitrogen determination apparatus (Kjeltec 2100; Foss, Hillerod, Denmark). Ether extract (EE) were determined in accordance with AOAC (2005) method 920.39 using an AnkomXT15 Extractor (Ankom Technology, Fairport, NY, USA). An Ankom fiber analyzer (Ankom Technology, USA) was used to examine acid detergent fiber (ADF), and neutral detergent fiber (NDF) was analyzed by following Association of Official Analytical Chemists (AOAC, 2005; method 973.18) [17]. The concentration of ammonia-N (NH_3_-N) was analyzed by using the indophenol blue method [18]. Acetate (AA) and butyrate (BA) contents were determined by using a gas chromatography device (GC-14B; Shimadzu, Kyoto, Japan; film thickness of the capillary column, 30 m×0.32 mm×0.25 mm; column temperature, 110°C; injector temperature, 180°C; and detector temperature, 180°C) and lactic acid (LA) analysis was performed by using a spectrophotometric method [19]. Total digestible nutrients (TDN), 30 h *in vitro* dry matter digestibility (IVDMD_30 h_), and 30 h *in vitro* neutral detergent fiber digestibility (IVNDFD_30 h_) were evaluated by Hangzhou Aike Testing Technology Co., Ltd. (Hangzhou, China).

#### Relative feed value

The relative feed value (RFV) was developed by the Hay Marketing Task Force of the American Forage and Grassland Council. RFV is calculated from an estimat of ADF (% DM) and NDF (% DM). The formula for calculating RFV is:


RFV (%)=93×(88.9-0.779×ADF)/NDF

### Statistical analysis

Data from this study were analyzed by one-way analysis of variance using SPSS software (version 23) according to the following model: Y_ij_ = μ+D_i_+C_j_+e_ij_, where Y_ij_ is the observation of dependent variables; μ is the overall mean; D_i_ represents the fixed effect of treatment; C_j_ is the random corn effect and e_ij_ is the residual error for an observation. Differences among treatment means were classified using Duncan’s post hoc test for multiple comparisons. Results were considered statistically significant at p<0.05.

## RESULTS

### The plant height, stem diameter, yield and relative feed value of corn with biogas slurry application

**Figure 1:**
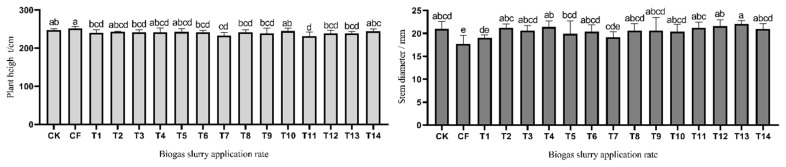
Effects of different biogas slurry application rate on the height and stem diameter of corn. CK, no nitrogen fertilizer; CF, chemical synthetic fertilizer; T1, 25% biogas slurry nitrogen replacement; T2, 50% biogas slurry nitrogen replacement; T3, 75% biogas slurry nitrogen replacement; T4, 100% biogas slurry nitrogen replacement; T5, 125% biogas slurry nitrogen replacement; T6, 150% biogas slurry nitrogen replacement; T7, 175% biogas slurry nitrogen replacement; T8, 200% biogas slurry nitrogen replacement; T9, 250% biogas slurry nitrogen replacement; T10, 300% biogas slurry nitrogen replacement; T11, 350% biogas slurry nitrogen replacement; T12, 400% biogas slurry nitrogen replacement; T13, 450% biogas slurry nitrogen replacement; T14, 500% biogas slurry nitrogen replacement. ^a–d^ Different lowercase letters indicate significant difference among the different fertilizer treatments at the 0.05 level.

**Figure 2:**
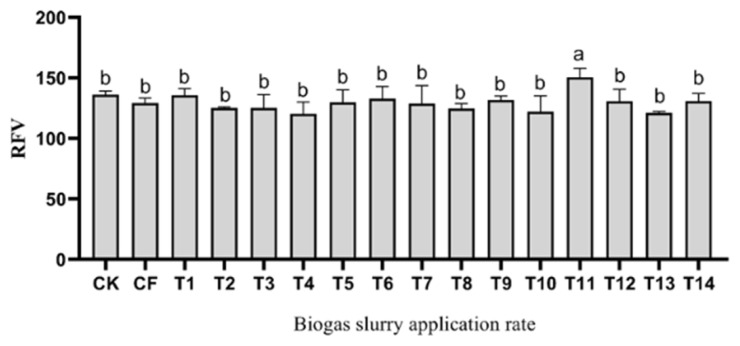
Effects of different biogas slurry application rate on relative feeding value of corn silage. CK, no nitrogen fertilizer; CF, chemical synthetic fertilizer; T1, 25% biogas slurry nitrogen replacement; T2, 50% biogas slurry nitrogen replacement; T3, 75% biogas slurry nitrogen replacement; T4, 100% biogas slurry nitrogen replacement; T5, 125% biogas slurry nitrogen replacement; T6, 150% biogas slurry nitrogen replacement; T7, 175% biogas slurry nitrogen replacement; T8, 200% biogas slurry nitrogen replacement; T9, 250% biogas slurry nitrogen replacement; T10, 300% biogas slurry nitrogen replacement; T11, 350% biogas slurry nitrogen replacement; T12, 400% biogas slurry nitrogen replacement; T13, 450% biogas slurry nitrogen replacement; T14, 500% biogas slurry nitrogen replacement; RFV, relative feed value. ^a,b^ Different lowercase letters indicate significant difference between the different fertilizer treatments at the 0.05 level.

Plant height and stem diameter are important components for determining biomass production in corn. Figure 1 shows that the plant height and stem diameter of corn varied at different levels of biogas slurry treatment. The application of different levels of biogas slurry was no different (p>0.05) among the T1 to T9 and T11 to T13 groups, and the results show that a high proportion of biogas slurry application has an inhibitory effect on the plant height of corn. The plant height of corn was higher (p<0.05) in the CK and CF groups than in the T11 to 13 groups. The stem diameter of corn was higher (p<0.05) in the high-level biogas slurry treatments (T11 to T13 groups) than in the low level biogas slurry treatments (T1 group). The biogas slurry groups had higher (p< 0.05) stem diameters than the CF groups except for the T1 and T7 groups. Table 1 shows the fresh weight of the biomass yield of corn under different levels of biogas slurry treatment. Regarding stem weight, leaf weight and ear weight of corn, there were no differences (p>0.05) among the biogas slurry applications from the T1 to T10 groups, and compared to the T8 group, T12 and 13 groups had higher (p<0.05) leaf weights. The corn treated with T13 displayed the highest fresh weight, which was 80.37 t/hm^2^. In the case of RFV, as shown in Figure 2, the corn silage at T11 had the highest RFV of all the corn silages.

### The fermentation quality of corn silage with biogas slurry application

The fermentation quality of the corn silage in the different biogas slurry treatment groups is presented in Table 2. Biogas slurry treatments had no effects (p>0.05) on pH and NH_3_-N. The pH values in the study varied from 3.80 to 3.90, which were within the ideal range for corn silage. The LA contents of corn silage samples were higher (p<0.05) for T11 (2.15% DM) than for the CF and T1 groups, but there was no difference (p>0.05) from the other biogas slurry treatment groups. In the case of AA content, the highest AA content was in T13 (1.037% DM). For the BA contents, there were no statistical differences among the biogas slurry treatment groups compared to the CF group.

### Nutritional values of corn silage with biogas slurry application

The nutrient content of corn silage in the different biogas slurry treatment groups is presented in Table 3. The content of CP was no different (p>0.05) among the biogas slurry application groups, but the CP content was higher (p<0.05) for T5 (10.85% DM) than for the CK group. The EE content of T5 (3.52% DM) was higher (p<0.05) than that of CF (2.89% DM), T1 (2.98% DM), T3 (2.90% DM), T8 and T12 (both with 2.91% DM). In the case of ADF and NDF content, the differences (p<0.05) were observed among the treatment groups. The lowest ADF content was in T11 (23.32% DM). Among the biogas slurry application groups, the T4 group had the highest NDF content (47.75% DM), and the T11 groups also had the lowest NDF content (40.57% DM). The ADF content was not different (p>0.05) among the biogas slurry application groups except for the T4 and T11 groups. In addition, the starch content of the T11 group was higher (p<0.05) than that of the other treatment groups.

### The digestibility and total digestible nutrients in corn silage with biogas slurry application

The digestibility and TDN of corn silage in the different biogas slurry treatment groups are presented in Table 4. The IVDMD_30 h_ of the T11 group was higher (p<0.05) than that of the CF, T1, T2, T4, and T10 groups. There was no difference (p>0.05) in the IVNDFD_30 h_ among the biogas slurry application groups except for the T2 group. The IVNDFD_30 h_ was higher (p<0.05) in the T6, T7, and T13 groups than in the T2 groups. Additionally, for the TDN, that of T12 group was highest compared to other treatments.

## DISCUSSION

Biogas slurry, as a high-quality organic fertilizer, is rich in nitrogen, phosphorus, potassium and humic acid, organic matter, amino acids, growth hormones, antibiotics, trace elements and other nutrients, which can meet the requirements for normal crop production and improve the growth parameters of corn [20]. In addition, as a kind of biofertilizer with abundant nutrients, biogas slurry has also been reported to increase yields in tuberosum [21], Spinacia oleracea and Capsicum annuum [22] compared with the application of synthetic fertilizer alone. However, in this study, there was no difference in the fresh yield of corn with biogas slurry application, which is consistent with the results of Wentzel et al [23], which reported no increase in the biomass yield of Italian ryegrass with use of excess biogas slurry. The reason may be that excessive biogas slurry application led to a high soil carbon to nitrogen ratio in the early stage, and soil microorganisms and crops competed for nitrogen, which affected crop growth [24].

Ensiling is a fermentation process driven by lactic acid bacteria, which ferment water-soluble carbohydrates (WSC) into organic acids (mainly lactic acid) in an anaerobic environment. As a result, the pH decreases, and forage is preserved. Studies of the effects of the nitrogen application rate on the fermentation quality of whole corn, forage sorghum, perennial ryegrass and other gramine forage silage showed that the nitrogen application rate had a certain effect on the silage pH, LA, AA, NH_3_-N and other indices, but the trend for each was different. Most studies indicated that with an increase in the nitrogen application rate, the content of NH_3_-N tended to increase, while the contents of pH, LA, and AA were different among grass species and among different experiments, and the results showed no consistency [25]. pH is a crucial factor in silage preservation [26]. It has been demonstrated that destructive fermentation is inhibited as pH declines [27]. Corn silage is generally required to have a pH value of 3.6 to 4.2, with an ideal range of 3.8 to 3.9. In the present study, the pH value of the biogas slurry application group was 3.8 to 3.9. Appropriate pH promotes beneficial microbial activity, and silage is well preserved even after storage for a year. The content of organic acids can reflect the quality of silage in the fermentation process. Organic acid content is related to feed intake, especially lactic acid content, which has a direct effect on palatability. This study revealed that the content of LA in the biogas slurry application groups was generally higher than that in the CF groups. The aerobic stability of silage is important because it relates to the safety and quality of the preserved forage upon exposure to air during storage and feeding. Schmidt and Kung [28] reported that accumulation of acetic acid was the main reason for the improved aerobic stability of silage. In this study, the content of acetic acid was no different between the biogas slurry application groups and the CF groups. However, the T13 group had the highest AA content. BA is the product of Clostridium fermentation, and a lower the concentration is better. In this study, there was no difference in the content of BA among the treatments, indicating that biogas slurry had no negative effect on the fermentation quality of corn silage.

The nutritional quality of corn is an important index to evaluate the quality of silage. The CP and crude fiber contents of forage are the most important indicators of forage quality. Generally, they have a direct effect on milk production in dairy animals and body growth in cattle. In general, increasing the amount of nitrogen applied can increase the CP content, decrease the fiber content, and improve the nutritional value of herbage. It was reported that the content of CP increases with increasing nitrogen fertilizer dose in Zamboo grass [29]. Similar results were also reported in another study: as the nitrogen application rate rose, the contents of crude fiber, NDF and ADF decreased, and the CP and WSC contents and RFV increased [16]. However, in this study, the application of biogas slurry did not increase the content of CP in corn, which may be attributed to the slow mineralization of organic fertilizer. Additionally, the low supply of available nutrients within the slow-acting organic fertilizer can affect the synthesis of nutrients such as CP and WSC in herbage. The contents of NDF and ADF in feed play an important role in maintaining the normal fermentation functions of an herbivore’s rumen. The NDF content is negatively correlated with the pH value of the rumen, but excessive ADF content will affect the palatability of forage, having a negative effect on DM intake [30]. In this study, the contents of ADF and NDF in the T11 group were lowest among the biogas slurry treatments. The main component of forage stalks is cellulose, and the contents of NDF and ADF are high when forage stalks grow rapidly. Under the application of T11 biogas slurry, the growth rate of forage stalk decreased, and the content of NDF and ADF decreased, which was consistent with the observation that the T11 group had the lowest plant height. The digestible nutrient content of forage is related to the conversion rate of livestock products and is an important indicator for evaluating the nutritional value of forage. In this study, compared with the CF group, the T11 and T13 groups had higher IVDMD_30 h_, IVNDFD_30 h_, and TDN. This shows that at this application rate, biogas slurry application has the potential to improve forage digestibility and forage quality.

## CONCLUSION

Biogas slurry, a byproduct of biogas production generated from the anaerobic digestion of animal waste and crop residues, is often considered a substitute to reduce mineral fertilizer input. The results of the present study showed that biogas slurry application improved the stem diameter and RFV of corn silage in the T13 and T11 treatment groups. Moreover, the fermentation quality of corn silage was improved due to an increase in LA content in comparison with the chemical synthetic fertilizer group. The CP contents of corn silage had no obvious change with increasing biogas slurry application. However, the forage quality index of ADF had a decrease compared with the chemical synthetic fertilizer group. In addition, higher IVDMD_30 h_ and IVNDFD_30 h_ were observed in the T11 and T13 groups than in the CF group. Based on these results, it was concluded that the optimum biogas slurry application for corn was 350% to 450% under the present experimental conditions.

## Figures and Tables

**Table 1:** Effects of different biogas slurry application rate on the fresh yields of corn

Treatment	Stem weight (g)	Leaf weight (g)	Ear weight (g)	Fresh yields (t/hm^2^)
CK	309.50^c^	135.50^b^	226.50^b^	67.23^b^
CF	339.60^bc^	150.80^ab^	294.80^a^	78.62^a^
T1	352.40^abc^	153.60^ab^	257.00^ab^	76.40^ab^
T2	350.67^bc^	145.00^ab^	260.00^ab^	75.66^ab^
T3	341.80^bc^	142.00^ab^	274.40^ab^	75.92^ab^
T4	366.00^ab^	150.00^ab^	228.20^b^	74.51^ab^
T5	338.33^bc^	146.67^ab^	250.33^ab^	73.63^ab^
T6	344.75^bc^	144.75^ab^	266.25^ab^	75.67^ab^
T7	338.83^bc^	144.17^ab^	239.17^b^	72.31^ab^
T8	340.33^bc^	135.00^b^	254.33^ab^	73.06^ab^
T9	349.80^bc^	138.60^ab^	251.40^ab^	74.07^ab^
T10	345.00^bc^	149.83^ab^	230.50^b^	72.62^ab^
T11	364.17^ab^	153.17^ab^	258.33^ab^	77.66^a^
T12	370.50^ab^	157.33^a^	267.67^ab^	79.65^a^
T13	399.00^a^	156.67^a^	247.00^ab^	80.37^a^
T14	356.33^abc^	148.00^ab^	254.33^ab^	75.96^ab^
SEM	21.131	8.007	21.360	4.063
p-value	0.078	0.140	0.174	0.255

CK, no nitrogen fertilizer; CF, chemical synthetic fertilizer; T1, 25% biogas slurry nitrogen replacement; T2, 50% biogas slurry nitrogen replacement; T3, 75% biogas slurry nitrogen replacement; T4, 100% biogas slurry nitrogen replacement; T5, 125% biogas slurry nitrogen replacement; T6, 150% biogas slurry nitrogen replacement; T7, 175% biogas slurry nitrogen replacement; T8, 200% biogas slurry nitrogen replacement; T9, 250% biogas slurry nitrogen replacement; T10, 300% biogas slurry nitrogen replacement; T11, 350% biogas slurry nitrogen replacement; T12, 400% biogas slurry nitrogen replacement; T13, 450% biogas slurry nitrogen replacement; T14, 500% biogas slurry nitrogen replacement; SEM, standard error of the mean.

a–c Different lowercase letters indicate significant differences (p<0.05) between different fertilization.

**Table 2:** Effects of different biogas slurry application rate on the fermentation quality of corn silage

Treatment	pH	NH_3_-N (%)	LA (%)	AA (%)	BA (%)
CK	3.80	0.13	1.99^ab^	0.88^ab^	<0.01
CF	3.87	0.123	1.659^b^	0.89^ab^	<0.01
T1	3.83	0.12	1.66^b^	0.97^ab^	ND
T2	3.83	0.14	1.71^ab^	0.92^ab^	ND
T3	3.90	0.13	1.78^ab^	1.03^a^	0.01
T4	3.90	0.14	1.81^ab^	0.94^ab^	0.01
T5	3.87	0.14	1.86^ab^	0.98^ab^	0.01
T6	3.80	0.11	1.89^ab^	0.84^b^	0.01
T7	3.87	0.12	1.79^ab^	0.93^ab^	0.01
T8	3.73	0.11	1.92^ab^	0.87^ab^	0.01
T9	3.87	0.12	1.75^ab^	0.97^ab^	<0.01
T10	3.83	0.12	1.83^ab^	0.96^ab^	ND
T11	3.90	0.12	2.15^a^	0.87^ab^	<0.01
T12	3.77	0.10	1.87^ab^	0.85^b^	<0.01
T13	3.77	0.14	1.88^ab^	1.04^a^	0.01
T14	3.83	0.11	1.93^ab^	0.98^ab^	<0.01
SEM	0.086	0.022	0.187	0.074	0.008
p-value	0.763	0.876	0.564	0.217	0.814

LA, lactic acid; AA, acetic acid; BA, butyric acid; CK, no nitrogen fertilizer; CF, chemical synthetic fertilizer; ND, not detected; T1, 25% biogas slurry nitrogen replacement; T2, 50% biogas slurry nitrogen replacement; T3, 75% biogas slurry nitrogen replacement; T4, 100% biogas slurry nitrogen replacement; T5, 125% biogas slurry nitrogen replacement; T6, 150% biogas slurry nitrogen replacement; T7, 175% biogas slurry nitrogen replacement; T8, 200% biogas slurry nitrogen replacement; T9, 250% biogas slurry nitrogen replacement; T10, 300% biogas slurry nitrogen replacement; T11, 350% biogas slurry nitrogen replacement; T12, 400% biogas slurry nitrogen replacement; T13, 450% biogas slurry nitrogen replacement; T14, 500% biogas slurry nitrogen replacement; SEM, standard error of the mean.

a,b Different lowercase letters indicate significant differences (p<0.05) between different fertilization.

**Table 3:** Effects of different biogas slurry application rate on the nutrient content of corn silage

Treatments	Starch (% DM)	CP (% DM)	EE (% DM)	Ash (% DM)	ADF (% DM)	NDF (% DM)
CK	28.67^ab^	9.63^b^	3.09^abc^	4.77^bc^	25.16^ab^	43.60^bcd^
CF	27.63^abc^	9.79^ab^	2.899^c^	5.19^abc^	26.35^a^	45.07^abc^
T1	28.03^abc^	10.00^ab^	2.989^bc^	4.76^bc^	26.04^a^	43.08^cd^
T2	27.23^abc^	9.80^ab^	3.05^abc^	5.23^abc^	27.28^a^	45.87^abc^
T3	25.93^abcd^	10.29^ab^	2.90^c^	4.75^bc^	26.99^a^	46.21^abc^
T4	25.10^bcd^	10.46^ab^	3.15^abc^	5.16^abc^	27.36^a^	47.75^a^
T5	24.80^bcd^	10.85^a^	3.53^a^	4.94^abc^	26.45^a^	44.83^abc^
T6	24.60^bcd^	10.00^ab^	3.08^abc^	5.24^abc^	25.705^ab^	44.33^abc^
T7	25.00^bcd^	10.39^ab^	3.40^abc^	5.58^a^	25.925^a^	45.70^abc^
T8	24.73^bcd^	9.84^ab^	2.91^c^	5.37^ab^	26.72^a^	46.46^abc^
T9	24.97^bcd^	10.24^ab^	3.21^abc^	4.49^c^	25.96^a^	44.51^abc^
T10	25.97^abcd^	10.14^ab^	3.24^abc^	5.08^abc^	27.64^a^	46.87^abc^
T11	30.93^a^	9.83^ab^	3.07^abc^	4.75^bc^	23.32^b^	40.57^d^
T12	21.93^d^	9.89^ab^	2.91^c^	4.98^abc^	26.10^a^	44.85^abc^
T13	23.27^cd^	10.47^ab^	3.45^ab^	5.39^ab^	27.57^a^	47.12^ab^
T14	27.93^abc^	9.78^ab^	3.23^abc^	4.74^bc^	26.26^a^	44.54^abc^
SEM	2.179	0.456	0.214	0.335	1.133	1.670
p-value	0.037	0.396	0.088	0.126	0.087	0.027

DM, dry matter; CP, crude protein; EE, ether extract; ADF, acid detergent fiber; NDF, neutral detergent fiber; CK, no nitrogen fertilizer; CF, chemical synthetic fertilizer; T1, 25% biogas slurry nitrogen replacement; T2, 50% biogas slurry nitrogen replacement; T3, 75% biogas slurry nitrogen replacement; T4, 100% biogas slurry nitrogen replacement; T5, 125% biogas slurry nitrogen replacement; T6, 150% biogas slurry nitrogen replacement; T7, 175% biogas slurry nitrogen replacement; T8, 200% biogas slurry nitrogen replacement; T9, 250% biogas slurry nitrogen replacement; T10, 300% biogas slurry nitrogen replacement; T11, 350% biogas slurry nitrogen replacement; T12, 400% biogas slurry nitrogen replacement; T13, 450% biogas slurry nitrogen replacement; T14, 500% biogas slurry nitrogen replacement; SEM, standard error of the mean.

a–d Different lowercase letters indicate significant differences (p<0.05) between different fertilization.

**Table 4:** Effects of different biogas slurry application rate on the *in vitro* dry matter digestibility of corn silage

Treatments	30 h *in vitro* dry matter digestibility (%)	30 h *in vitro* neutral detergent fiber digestibility (%)	Total digestible nutrients (%)
CK	80.67^abc^	55.67^abc^	70.33^abc^
CF	79.00^bc^	53.33^bc^	69.00^bc^
T1	80.00^bc^	54.00^abc^	70.00^abc^
T2	78.00^c^	51.67^c^	69.00^bc^
T3	80.67^abc^	57.67^abc^	69.00^bc^
T4	79.67^bc^	57.33^abc^	70.00^abc^
T5	80.67^abc^	57.33^abc^	70.00^abc^
T6	82.33^ab^	60.00^a^	69.67^abc^
T7	81.33^abc^	60.00^a^	69.67^abc^
T8	80.33^abc^	58.00^ab^	69.00^bc^
T9	81.67^ab^	59.00^ab^	69.67^abc^
T10	79.67^bc^	57.00^abc^	70.67^abc^
T11	83.67^a^	59.67^ab^	72.33^a^
T12	81.33^abc^	58.33^ab^	67.67^c^
T13	81.33^abc^	60.33^a^	70.00^abc^
T14	81.67^ab^	59.00^ab^	71.67^ab^
SEM	1.477	2.653	1.318
p-value	0.107	0.070	0.207

CK, no nitrogen fertilizer; CF, chemical synthetic fertilizer; T1, 25% biogas slurry nitrogen replacement; T2, 50% biogas slurry nitrogen replacement; T3, 75% biogas slurry nitrogen replacement; T4, 100% biogas slurry nitrogen replacement; T5, 125% biogas slurry nitrogen replacement; T6, 150% biogas slurry nitrogen replacement; T7, 175% biogas slurry nitrogen replacement; T8, 200% biogas slurry nitrogen replacement; T9, 250% biogas slurry nitrogen replacement; T10, 300% biogas slurry nitrogen replacement; T11, 350% biogas slurry nitrogen replacement; T12, 400% biogas slurry nitrogen replacement; T13, 450% biogas slurry nitrogen replacement; T14, 500% biogas slurry nitrogen replacement; SEM, standard error of the mean.

a–c Different lowercase letters indicate significant differences (p<0.05) between different fertilization.
